# Biomarker Insights Into Brain Health, Aging, and Healthspan

**DOI:** 10.1093/geroni/igaf122.2082

**Published:** 2025-12-31

**Authors:** Breno Diniz, Qu (Teresa) Tian

## Abstract

There has been a surge in the novel biomarker approach to identify early predictors of diseases, understand multiple disease etiology, and, more importantly, the effect of biological aging on health and disease dynamics. In this symposium, we will present novel biomarker data to reveal novel pathways related to brain health, dementia, and biomarker signatures of longevity and healthspan. This symposium features 5 presentations of recently conducted studies using biomarker data from US and European cohorts. First, Dr. Diniz will present recent results suggesting that a history of major depression is causally associated with biological aging acceleration using Mendelian randomization approaches. Second, Dr. Tian will present data about developing a novel biomarker composite score based on eye-movement measures and its association with age-related cognitive and mobility decline. Third, Dr. Evans uses data from the Longevity Consortium to examine metabolomic signatures of human aging and longevity. Firth, Dr. Liu shows a novel protein-based signature predictive of cumulative frailty index, the proteomic frailty index (pFI), and examines the association of pFI with multiple health indicators in three independent cohorts, including physical, clinical and cognitive measures, dementia, and mortality. Finally, Dr. Kuo will show the development of a healthspan proteomic score (HPS) to identify proteomic signatures of healthspan and its association with mortality and multiple age-related adverse health outcomes. Based on the symposium presentations, we seek to generate discussions of how biomarkers can aid the understanding of novel risk factors for accelerated biological aging, dementia risk, and healthspan.

